# Variables in the effect of land use on soil extrapore enzymatic activity and carbon stabilization

**DOI:** 10.1038/s41467-020-19900-9

**Published:** 2020-12-22

**Authors:** Melanie M. Glenn

**Affiliations:** Department of Plant Sciences, Department of Biosystems Engineering and Soil Sciences, UT Institute of Agriculture, The University of Tennessee, Knoxville, TN 37996 USA

**Keywords:** Carbon cycle, Microbial ecology, Solid Earth sciences

**Arising from** Kravchenko et al. *Nature Communications* 10.1038/s41467-019-11057-4

The recent study by Kravchenko et al.^1^ that was published in *Nature Communications* explores the relationship between microbial spatial footprints and soil carbon (C) storage capacity. The authors concluded that plant-stimulated soil pore formation is a major determinant of soil C sequestration rates, that diverse plant communities favor the development of 30–150 µm pores, and that pores in this size range are associated with higher enzymatic activity and thus higher C sequestration rates. However, these are cause-and-effect conclusions that are not supported by the data and research design that was implemented. The implications of this are that the study more clearly depicts effects of land use on various ecosystems and its association with soil microbial spatial footprints, rather than a direct causal relationship as stated by the authors.

First, Kravchenko et al.^1^ suggest that 30–150 µm pores contain the most active microbial communities, resulting in higher rates of C processing and sequestration. However, this is only true for the monoculture systems and not for the two biodiverse ecosystems. The biodiverse native vegetation systems (poplar and native succession) were reported to contain the highest amount of 30–150 µm pores, and thus would be expected to show the highest rates of enzyme activity. However, the data in Fig. 4a of Kravchenko et al. contradict this conclusion. Distribution of enzymatic activity surrounding 30–150 µm pores is reported as less in the diverse native ecosystems than in the monoculture ecosystems. This represents a significant discrepancy between the data and the conclusions that were drawn.

Second, the authors also concluded that diverse plant communities favor development of 30–150 µm pores. However, due to the use of a non-experimental design, such cause-and-effect conclusions cannot be drawn due to uncontrolled variables^2,3^. Specifically, the presence of these pores cannot be uniquely attributed to the heterogeneity of the plant communities. While root growth is indeed a method of pore development, there are other major factors affecting porosity in soil: the movement of water and biota through the matrix, organic matter and mineral content, depth of surface and subsurface horizons, chemical reactions, historical management practices, and more^4^. These properties may vary dramatically from one studied ecosystem to another, even among similar climates and topography. It would be necessary to determine the degrees of influence of each of these factors and more before a conclusion can be reached regarding the effect of plant community diversity on pore development. The whole-system processes and attributes are neither controlled nor measured by the authors, so only conclusions about associations and not effects are warranted. The study is a comparatively clearer look at the effects of land use on various ecosystems and its association with soil microbial spatial footprints.

A more limited replacement conclusion is proposed: “The factors of ecosystem biodiversity, microbial biomass, soil carbon partitioning, porosity, and extrapore enzymatic activity are all moderately associated with one other across ecosystems, with enzymatic activity level at its highest in the soil surrounding 30 µm pores in poplar systems and 30–150 µm pores in monocultures.”

The study has many major strengths, including the technical feat of combining X-ray micro-tomography for pore mapping with enzyme spatial distribution mapping, a 9-year duration for the tracking of soil properties in five different systems, excellent sampling and methodology, advanced statistical analysis, and an experimental design for the fresh nutrient input incubation portion of the study. Nonetheless, for these reasons stated above, the primary conclusion that 30–150 µm pores in diverse plant communities have a greater soil C storage capacity is not sufficiently supported to warrant adoption into soil conservation and regenerative agriculture approaches.

## Reporting summary

Further information on research design is available in the Nature Research Reporting Summary linked to this article.

## Supplementary information

Reporting Summary

## Data Availability

Data sharing not applicable to this article as no datasets were generated or analyzed during the current study.
